# High-level production of *Aspergillus niger* prolyl endopeptidase from agricultural residue and its application in beer brewing

**DOI:** 10.1186/s12934-023-02087-1

**Published:** 2023-05-04

**Authors:** Minglu Liu, Meng Hu, Hui Zhou, Zhiyang Dong, Xiuzhen Chen

**Affiliations:** 1 State Key Laboratory of Microbial Resources, Institute of Microbiology, Chinese Academy of Sciences, Beijing, 100101 China; 2 University of Chinese Academy of Sciences, Beijing, 100049 China

**Keywords:** *Trichoderma reesei*, Prolyl endopeptidase, Corn cob, Agricultural residues, *Aspergillus niger*, Gluten, Beer

## Abstract

**Background:**

Prolyl endopeptidase from *Aspergillus niger* (AN-PEP) is a prominent serine proteinase with various potential applications in the food and pharmaceutical industries. However, the availability of efficient and low-cost AN-PEP remains a challenge owing to its low yield and high fermentation cost.

**Results:**

Here, AN-PEP was recombinantly expressed in *Trichoderma reesei* (rAN-PEP) under the control of the *cbh1* promoter and its secretion signal. After 4 days of shaking flask cultivation with the model cellulose Avicel PH101 as the sole carbon source, the extracellular prolyl endopeptidase activity reached up to 16.148 U/mL, which is the highest titer reported to date and the secretion of the enzyme is faster in *T. reesei* than in other eukaryotic expression systems including *A. niger* and *Komagataella phaffii*. Most importantly, when cultivated on the low-cost agricultural residue corn cob, the recombinant strain was found to secret a remarkable amount of rAN-PEP (37.125 U/mL) that is twice the activity under the pure cellulose condition. Furthermore, treatment with rAN-PEP during beer brewing lowered the content of gluten below the ELISA kit detection limit (< 10 mg/kg) and thereby, reduced turbidity, which would be beneficial for improving the non-biological stability of beer.

**Conclusion:**

Our research provides a promising approach for industrial production of AN-PEP and other enzymes (proteins) from renewable lignocellulosic biomass, which provides a new idea with relevant researchers for the utilization of agricultural residues.

**Supplementary Information:**

The online version contains supplementary material available at 10.1186/s12934-023-02087-1.

## Background

Prolyl endopeptidase (PEP, EC 3.4.21.26), also called proline oligopeptidase, is a ubiquitous enzyme that preferentially cleaves peptide bonds at the carboxyl-terminal side of the internal proline residues. The prolyl endopeptidase from the food-grade fungus *Aspergillus niger* (AN-PEP; DSM, Heerlen, Netherlands) was originally characterized for its ability to debitter proline-rich casein hydrolysates [1]. It is currently a key food processing enzyme used to prevent chill-haze in beer and brew gluten-free foods including beer and bread [1–4] by removing proline-rich peptides and proteins such as gluten. Nowadays, a strict lifelong gluten-free diet is the only effective treatment for celiac disease (CD), a high-prevalence, auto-immune disorder triggered by gluten intake. Additionally, different from the bacterial PEPs derived from *Flavobacterium meningosepticum* (FM-PEP) [5–7], *Sphingomonas capsulate* (SC-PEP) [8] *Myxococcus xanthus* (MX-PEP) [9], and *Chryseobacterium taeanense* (CT-PEP) [10], which only hydrolyzes oligopeptides of no more than 33 amino acids [9, 11], presents optimal activity at pH 7.0–8.0 and abolishes the proteolytic capabilities in the acidic and proteolytic environment of stomach, AN-PEP with an acidic pH optimum [1, 12, 13] has the ability toward high-efficient hydrolysis of gluten-derived celiac disease-triggering immunogenic peptides and intact gluten under acidic conditions [14], hence allowing early-on cleavage and degradation of gluten present in the stomach. Due to these unique and excellent properties, AN-PEP has also been explored as an enzyme supplement Tolerase^®^ G (www.dsm.com/tolerase-g) for the conventional dietary treatment of CD. With the increasing demand for high-quality gluten-free foods and oral enzyme supplements, the production of prolyl endopeptidase has become an important concern.

FM-PEP, SC-PEP and MX-PEP were recombinantly expressed in *E. coli*, yielding 1 mg/ L, 60 mg/ L and 30 mg/ L of active proteins, respectively [9]. Even though overproduction of MX- PEP was achieved (a yield of 0.25–0.4 g/ L purified protein) through fed-batch fermentation [15], *Flavobacterium meningosepticum*, *Myxococcus xanthus* and *Sphingomonas capsulate* belong to the pathogenic bacteria and hence are not good choices for applications in the food industry. Therefore, how to improve the production of AN-PEP from the food-grade microorganism is taking the centre stage. Until recently, AN-PEP was only produced by its natural producer *A. niger* at a relatively low yield (9.76 ± 0.06 U/kg after 168 h of solid-state fermentation), although process conditions were optimized to maximize the yield of the enzyme [16]. Recombinant expression offers a practical alternative to boost AN-PEP enzyme production. AN-PEP has been biotechnologically produced in the prokaryotic host *Escherichia coli* with inclusion body [17] and more efficiently in the eukaryotic cell factory *Pichia pastori* (*Komagataella phaffii*) through codon optimization and high cell density fermentation with a maximum yield of 1.89 U/ mL that was done in a 7 L fermenter using glycerol as the sole carbon source and methanol as an inducer [13]. So far, the efforts invested in PEP production have been very limited, and the industrial production of AN-PEP still faces various challenges, such as low yield and high-cost of fermentation. It is highly desirable to develop an efficient, sustainable, and safe system for AN-PEP production.

The filamentous fungus *Trichoderma reesei* is one of the most extraordinary producers of lignocellulose-degrading enzymes, of which cellobiohydrolase I (CBHI) is predominantly produced from the single-copy *cbh1* gene, which is strongly induced by cellulose, sophorose, and complex plant materials [18]. Meanwhile, due to its extraordinary production and secretion capacity and the generally regarded as safe (GRAS) status approved by the United States Food and Drug Administration (FDA) [19, 20], *T. reesei* has also emerged as a potent host for producing proteins of interest for research, pharmaceuticals, and industry [21]. Apart from these advantages for heterologous protein production, *T. reesei* has evolved the ability to decompose the most abundant lignocellulosic biomass, mainly agro-industrial residues, which represents a sustainable and cheaper alternative carbon source or inducer for microbial growth and enzyme production [22–24]. Hence, *T. reesei* may represent a promising option for efficient and cost-effective production of An-PEP derived from *A. niger*.

Here, we used the *cbh1* promoter, *cbh1* secretion signal, and the *cbh2* terminator to drive the expression of *AN-PEP* gene in *T. reesei* strain and screened the best-performing transformants by cultivation in media containing the model cellulose Avicel PH101. In addition, we investigated the potential of rAN-PEP production in *T. reesei* using sustainable raw materials as alternative carbon sources and inducers. Finally, the application of recombinant AN-PEP in beer brewing was evaluated by analyzing residual gluten.

## Materials and methods

### Strains, media, and cultivation conditions

*Escherichia coli* strain Trans1-T1 (Beijing TransGen Biotech Co., Ltd., China) was used for standard cloning.

*Trichoderma reesei* TU6 (*ATCC* MYA-256), a uridine auxotrophic strain, was used as the host to express the prolyl endopeptidase gene from *A. niger.* The *T. reesei* strains were cultivated on a minimal medium (MM) containing the appropriate carbon source or potato dextrose agar (PDA) plates. MM without peptone was prepared as described previously [18]. The pH of MM was adjusted to 5.1 ± 0.2 with NaOH. When necessary, 5 mM of uridine or 0.1% (V/V) of Triton X-100 was added.

For replacement experiments [25], *T. reesei* strains were grown in a 250-mL Erlenmeyer flask containing 50 mL MM with 2% glucose as sole carbon source at 28 °C with shaking at 200 rpm for 48 h, and washed in MM without a carbon source, followed by 96 h of growth in 50 mL MM containing a carbon source (Avicel cellulose, corn cob, wheat bran, or straw powder) or their different combinations. Cultures were sampled and centrifuged at 13,300 × *g* for 10 min. The supernatants were used for enzyme activity and protein concentration assays as well as for SDS-PAGE analysis.

### Construction of recombinant T. reesei strains bearing *Aspergillus niger* prolyl endopeptidase gene expression cassette (AN-PEP)

The expression vectors were constructed using the plasmid *pEASY*^®^-blunt simple (Novagen, TransGen, Beijing, China) as the backbone. The primer pair Fvector/ Rvector was designed to amplify *pEASY*^®^-blunt simple to generate a linearized plasmid for the construction of the expression vector. Briefly, the promoter and signal peptide of *cbh1* (Trire2 ID:123989), the terminator of *cbh2* (Trire2 ID:72567), and the marker gene *pyr4* (Trire2 ID:74020) were obtained via using the primer pairs Fcbh1/ Rcbh1, Fcbh2/ Rcbh2, and Fpyr4/ Rpyr4, respectively, to amplify the genomic DNA isolated from *T. reesei* QM9414 (ATCC26921) that is a mutant derived from the wild-type strain QM6a via two-round random mutagenesis and selection for enhanced cellulase production [26], ligated with the linearized *pEASY*^®^-blunt simple through a Clone Express^®^ MultiS One Step Cloning Kit (Vazyme, Nanjing, China), and then transformed into *E. coli* Trans1-T1. The resulting plasmid was designated pCBH12, which was subsequently linearized by PCR using the primer pair Fcbh12/ Rcbh12.

The *AN-PEP* gene (2047 bp) with introns was generated through using the FAN-PEP/ RAN-PEP primer pair to amplify the genomic DNA of *Aspergillus niger* CBS513.88. Its intronless derivative AN-PEP^*^, was synthesized according to the GenBank sequence (Accession number: CAK45422). Both versions of the *AN-PEP* gene were ligated with linearized pCBH12. The resulting expression vectors that were designated pCBH12-AN-PEP (*AN-PEP* gene) and pCBH12-ANPEP* (*AN-PEP* cDNA) included the *cbh1* promoter, *cbh1* signal peptide, *A. niger PEP* gene, *cbh2* terminator, and *pyr4* expression cassette (2.7 kb).

The *AN-PEP* expression vectors were transformed into *T. reesei* strain TU6 via the electroporation protocol as described by Schuster et al. [27], and 0.1% Triton X-100 was used as a colony restrictor. The transformants were selected on MM plates without uridine. PCR was used to confirm the chromosomal integration of the *AN-PEP* expression cassette, and the amplified PCR products were subjected to sequencing analysis.

All primers used in this study were listed in Additional file 1: Table. S1.

### Purification of prolyl endopeptidase

Culture supernatants were harvested by centrifugation at 13,300 × *g* for 10 min. All purification steps were performed at 4℃. Solid ammonium sulfate with 40% and 80% saturation was added in turn to the supernatant for 4 h on ice. The protein precipitated with ammonium sulfate was dissolved in 0.1 M citrate/disodium phosphate buffer (pH 5.0) and dialyzed in the same buffer overnight, followed by concentration with an Amicon Ultra 30,000 MWCO membrane (Millipore) and further purification with HiTrap DEAE FF column, as described by Xu et al. [28]. The collected fractions were prepared for enzymatic analysis and purity was determined using SDS-PAGE.

### Enzyme activity assays

Prolyl endopeptidase activity was determined via a colorimetric assay as described by Eden et al. [1] using the substrate benzyloxycarbonyl-glycine-proline-p-nitroanilide (Z-Gly-Pro-*p*NA, Bachem, Bubendorf, Switzerland), which releases nitroaniline that can be monitored at 410 nm. Briefly, Z-Gly-Pro-*p*NA was dissolved in 1,4-dioxane (40%, V/V in 0.1 M pH 5.0 citrate/disodium phosphate buffer). The reaction mixture (1 mL of 0.1 M citrate/disodium phosphate buffer (pH 5.0), 0.25 mL of 4 mM Z-Gly-Pro-*p*NA, 0.1 mL of enzyme solution) was incubated at 37 °C for 10 min, and 3 mL of 0.2 M anhydrous sodium carbonate was added to stop enzyme catalysis. The reaction products were determined spectrophotometrically at 410 nm. One unit of PEP activity was defined as the release of 1 μmol of *p*-nitroanilide per minute under specified conditions. To measure specific enzyme activities (units/ mg protein), the protein concentration was measured using a Modified Bradford Protein Assay Kit (Sangon Biotech, Shanghai, China) with bovine albumin as a standard.

The optimal temperature for recombinant prolyl endopeptidase activity was determined using the standard activity assay at a temperature range of 20–80 °C. To estimate the thermal stability, the purified protein was incubated for 1 h at different temperatures (4, 20, 30, 40, 50, 60, 70, and 80 °C). Then, the activity was determined as mentioned above at pH 4.0 and 37 °C. The effect of pH on rAN-PEP activity was analyzed at 37 °C in various pH buffers (0.1 M citrate/disodium phosphate buffer, pH 3.0–8.0). The pH stability of rAN-PEP was determined at 37 °C in 0.1 M citrate/disodium phosphate buffer (pH 4.0) after the rAN-PEP was incubated in 0.1 M KCl–HCl buffer (pH 1.0–2.0), 0.1 M citrate/disodium phosphate buffer (pH 3.0–9.0), and 0.05 M borate hydroxide buffer (pH 9.0–11.0) at room temperature for 1 h. For the optimization test, the catalytic activity at the optimal pH or temperature was used as the control and defined as 100%. For pH and thermal stabilities, the catalytic activity without pre-incubation was treated as the control and defined as 100%.

The effects of metal ions or the inhibitors on rAN-PEP activity were analyzed by adding each component (Na^+^, Ca^2+^, Zn^2+^, Mn^2+^, Fe^3+^, Cu^2+^, Ni^2+^, Co^2+^, Li^+^, Mg^2+^, K^+^, Fe^2+^, phenylmethylsulfonyl fluoride (PMSF), sodium dodecyl sulfate (SDS), and ethylene diamine tetraacetic Acid (EDTA), at 5 mM final concentration) to the purified enzyme solution and incubating for 1 h at room temperature. Subsequently, enzyme activity was determined under the standard conditions (37 °C, pH 4.0). For analysis of ethanol tolerance, the rAN-PEP was treated with 10–50% ethanol at 37 °C for 8 h, the residual enzyme activity was measured at 37 °C and pH 4.0. All experiments were conducted in triplicate. Enzyme activities were defined as relative values (%), and the control sample was defined as 100%.

### Measurement of gluten content

Gluten concentration in beer samples was measured using the RIDASCREEN^®^ Gliadin Competitive (Art. No. R7021, R-Biopharm, Germany), which has been approved as Association of Official Analytical Chemists (AOAC) Official Method of Analysis (OMA), to determine gluten content in fermented products [29]. According to the instruction manual of RIDASCREEN^®^ Gliadin Competitive, the limit of detection (LOD) is 2.3 mg gliadin/ kg food (approximately 4.6 mg gluten/ kg food), while the limit of quantification (LOQ) is 5.0 mg gliadin/ kg food (approximately 10.0 mg gluten/ kg food). Special software, the RIDA®SOFT Win.net, is available for constructing calibration curves and calculating gluten concentrations with measured absorbance.

### Quality analysis of beer treated with recombinant prolyl endopeptidase

The brewing ingredients (wort, hop, and brewer’s yeast) used in this study were kindly provided by Professor Feng-Yan Bai at the State Key Laboratory of Mycology at the Institute of Microbiology, Chinese Academy of Sciences. Beer treatment using rAN-PEP was performed as described by Di Ghionno et al. [30]. At the beginning of fermentation, the wort added with hop and yeast was equally divided into ten portions, and then 400 mL per portion was transferred into a 1 L stainless steel fermentation bottle equipped with one-way exhaust, meanwhile the broth was supplemented with different dosages of rAN-PEP: 0 μg/ mL (blank control group); 2.5, 5, 10, 20, 40, 80, and 120 μg/ mL (crude rAN-PEP supernatant); 2.5 μg/ mL (the purified rAN-PEP); and supernatant from the recipient strain TU6 (negative control group). Each trial was fermented in a top-fermented manner at 20 °C for 20 days, while maturation took place for 9 days at 1 ℃. The resulting unfiltered beer was stored at 4 °C for further analysis.

The maturated beer from blank control group was treated with different concentrations of purified rAN-PEP (0, 1, 2, 4, 8 ug/mL) at low temperature (9 °C) for 5 h. After the treatment, the gluten content in the beer was determined.

Beer quality parameters, including pH and total polyphenols were determined according to a previously reported method [31]. Briefly, pH measurements of the reference beer and rAN-PEP-treated beer were performed using a pH meter (FE20, Mettler-Toledo Instruments (Shanghai) Co., Ltd). Total polyphenol content was measured using a spectrophotometer (UV–vis spectrophotometer, LS5) at 600 nm, and was calculated as P = A600 × 820, where P is the total polyphenol concentration (mg/ L), A600 is the absorbance at 600 nm, and 820 is the conversion factor between absorbance and total polyphenol content. All measurements were conducted in triplicate.

## Results and discussion

### Generation of the best-performing recombinant strains

**Fig. 1:**
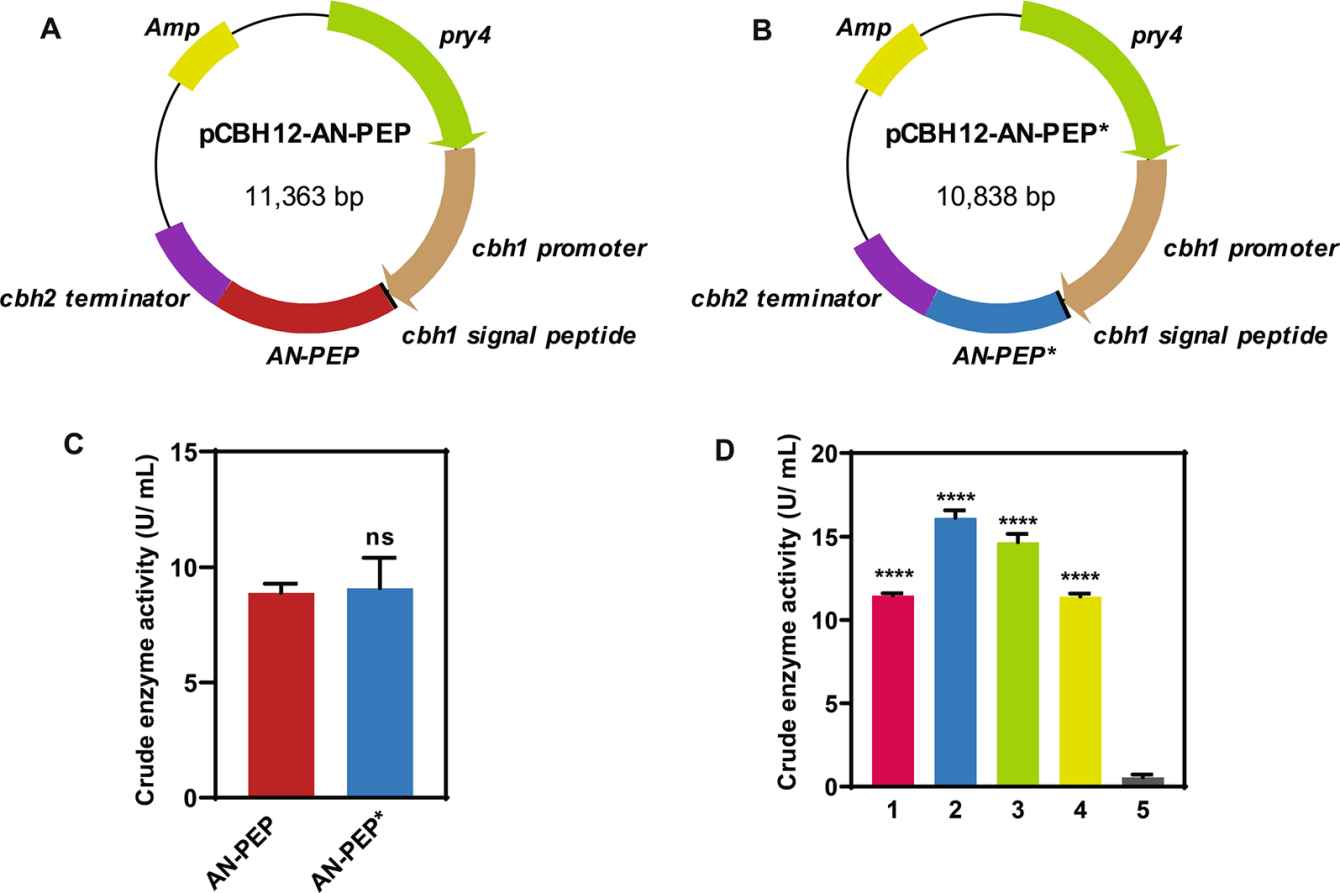
Plasmid profiles, and heterologous expression of prolyl endopeptidase in *T. reesei*. **A**, **B** Schematic drawing of plasmids pCBH12-AN-PEP and pCBH12-AN-PEP* bearing *cbh1* promoter, *cbh1* signal peptide, *A. niger PEP* gene, and *cbh2* terminator. *AN-PEP*: *A. niger PEP* gene with intron; AN-PEP*: *A. niger PEP* cDNA. **C** Activity analysis of prolyl endopeptidase secreted by transformants bearing pCBH12-AN-PEP and pCBH12-AN-PEP*. The error bar denotes the standard deviations (SD) of two biological replicates (ns: not significant). **D** Activity analysis of the positive transformants pCBH12-AN-PEP fermentation supernatants. The error bar denotes the standard deviations (SD) of three technical repeats (****p < 0.0001). 1–4: positive transformants 1–4; 5: *T. reesei* TU6

The *AN-PEP* gene from *A. niger* is 2040 bp in length, containing nine introns and 1,560 bp of the open reading frame with a theoretical molecular mass of 57.75 kDa. Since it is not clear whether the introns from *A. niger* could be efficiently spliced in *T. reesei* and exert positive effects on the expression of AN-PEP, the two constructs: intron-containing pCBH12-AN-PEP (Fig. 1A) and intronless pCBH12-AN-PEP* (Fig. 1B), were transformed into *T. reesei*, and the positive transformants identified by PCR were cultivated in MM with model cellulose Avicel PH101 as the sole carbon source. As shown in Fig. 1C, in terms of average enzyme activity, transformants bearing pCBH12-AN-PEP and pCBH12-AN-PEP* showed no statistical differences, whereas there were differences between individual transformants which might be due to different integration sites of the AN-PEP expression cassette, copy number, or their combination. Of particular note, the extracellular crude enzyme activity of prolyl endopeptidase was in the range of 11.415–16.148 U/ mL (Fig. 1D), which is the highest activity reported till now for prolyl endopeptidases derived from *A. niger* [13, 16, 17], and other microorganisms such as *Flavobacterium meningosepticum* [7], *Myxococcus xanthus* [15], *Sphingomonas capsulata* [8, 9, 15], *Xanthomonas* sp [32], *Aeromonas hydrophila* [33], *Pseudomonas* sp. KU-22 [34], *Aspergillus oryzae* [35], and *Flammulina velutipes* [36]. The best-performing recombinant strain bearing pCBH12-AN-PEP, designated as rAN-PEP, was chosen for further studies.

### A remarkable amount of rAN-PEP production from the agricultural residue corn cob

**Fig. 2:**
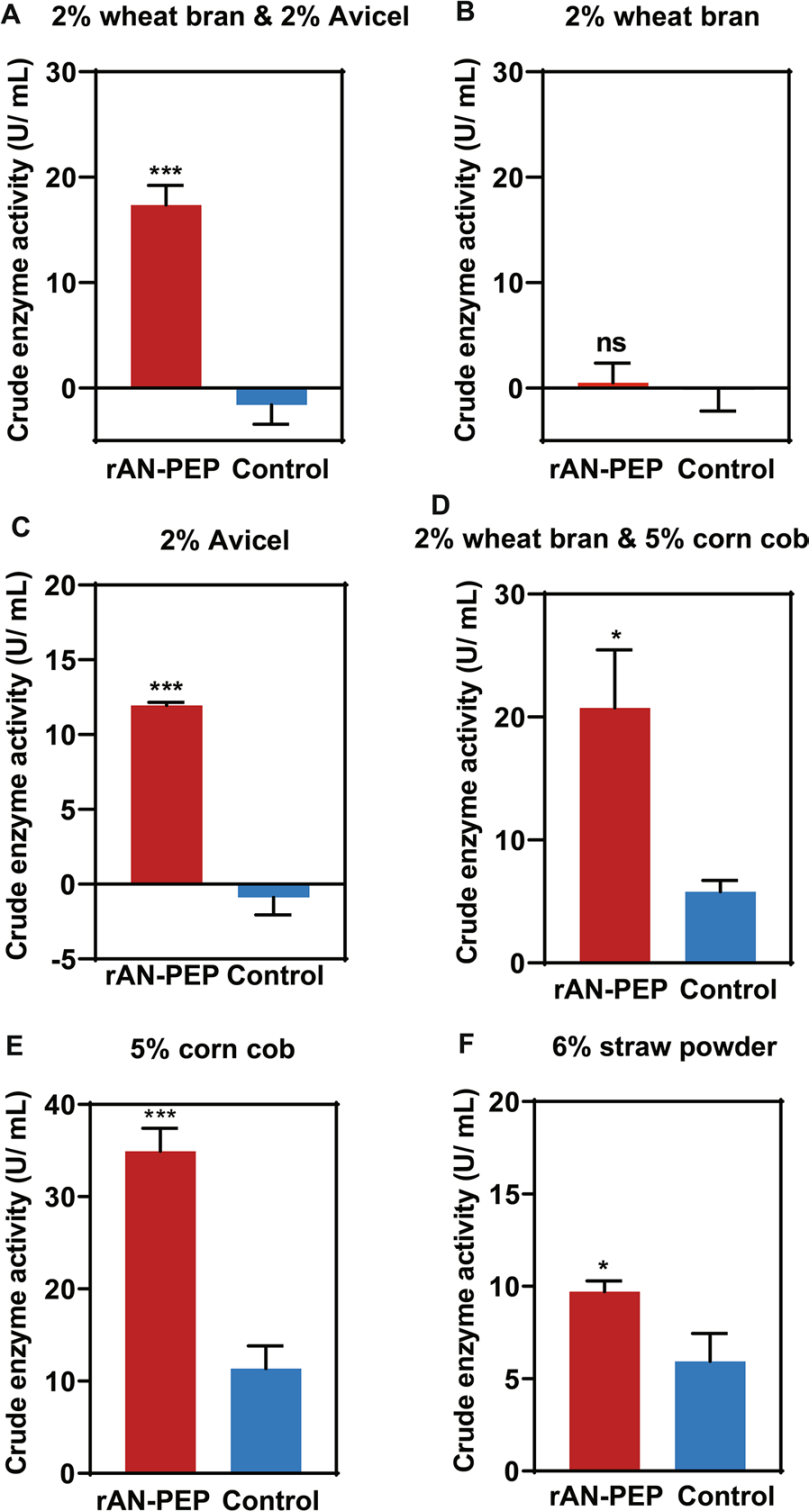
The activity analysis of extracellular prolyl endopeptidase under various carbon sources. *T. reesei* strain was pre-cultured in a minimal medium supplemented with 2% glucose for 48 h, then the mycelium was shifted to the MM containing two inducers [wheat bran & Avicel (**A**) and wheat bran & corn cob (**D**)] or a single inducer [wheat bran (**B**), Avicel PH101 (**C**), corn cob (**E**), and straw powder (**F**)]. After 4 days of fermentation, the supernatant was sampled for activity analysis. The error bar denotes the standard deviations (SD) of three technical repeats (*p < 0.05, ***p < 0.001, ns: not significant)

The lignocellulosic feedstock is the most abundant renewable resource, mainly agricultural and forestry residues, including wheat straw, rice straw, straw powder, corn cob, and corn stover, which are rich in cellulose, hemicellulose, and lignin [37]. Agricultural crop residues, with their cheap and easily accessible advantages, have gained increased attention from researchers over the last few decades [38]. However, most researchers have focused on saccharification of lignocellulosic biomass [39, 40]. To develop an efficient and sustainable production system for rAN-PEP, we investigated the potential of lignocellulosic biomass, such as wheat bran, corn cob, and straw powder, as a carbon source and inducer. According to a report by Fei Zhang et al. [40], either 2% wheat bran or 2% Avicel as the sole carbon source or the combination of the two biomasses was used to induce the expression of AN-PEP. As shown in Figs. 2A–C, the combination of 2% wheat bran and 2% Avicel was beneficial for increasing activity of rAN-PEP (approximately 45%) compared with the induction with 2% Avicel alone. However, 2% of wheat bran (11–13.8% cellulose [41]) alone did not induce the expression of AN-PEP. Based on this result, instead of expensive Avicel, 5% corn cob (32.3–45.6% cellulose in corn cob [42]) whose cellulose content is theoretically equal to 2% Avicel, together with 2% wheat bran, was added to MM to induce the expression of AN-PEP. Our results showed that the combination of 2% wheat bran and 5% corn cob caused an increase of rAN-PEP enzyme activity by 19.5% compared with the combination of 2% wheat bran and 2% Avicel (Fig. 2D), which indicates the excellent induction potential of corn cob in *T. reesei*. As expected, under the 5% corn cob induction condition, extracellular rAN-PEP activity reached 34.930 U/ mL (Fig. 2E), which is twice that in the initial induction with 2% wheat bran and 2% Avicel, whereas 6% straw powder that is approximately corresponding to 2% Avicel, could induce the expression of AN-PEP but with the titer at an average level (Fig. 2F).

### Enzymatic properties of prolyl endopeptidase

Through ammonium sulfate precipitation, dialysis, ultrafiltration and anion exchange chromatograph, the effective separation of rAN-PEP was achieved with the purification coefficient of 6.45 and the recovery of 44.98% (Additional file 1: Table. S2). And the obtained rAN-PEP showed relatively high homogeneity as indicated by SDS-PAGE (Additional file 1: Fig. S1) and hence was used for subsequent analysis.

**Fig. 3:**
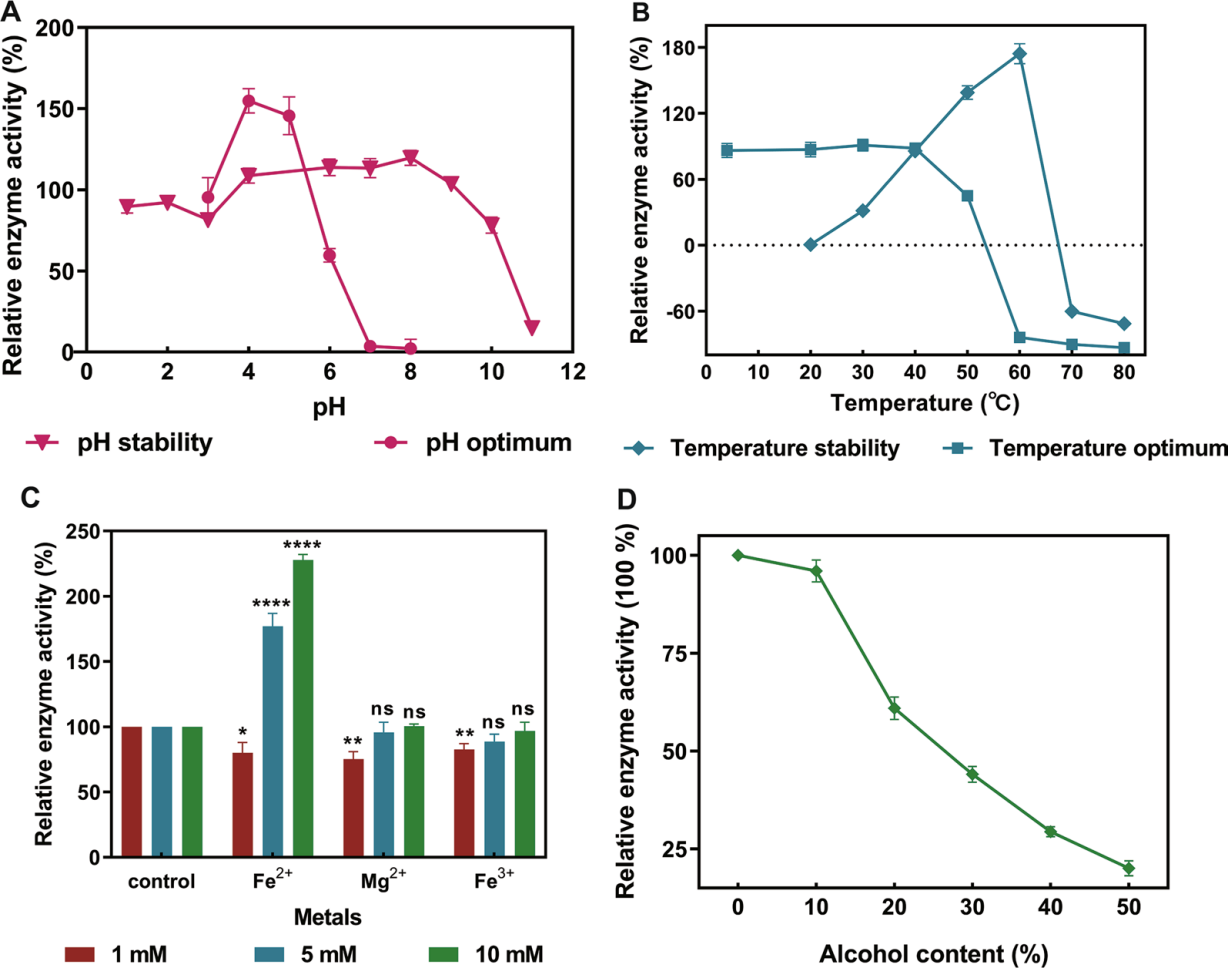
Enzymatic properties of recombinant prolyl endopeptidase. **A** The effect of pH on recombinant prolyl endopeptidase activity and stability. The pH optimum was determined between pH 3.0–8.0 at 37 ℃. The pH stability was determined by incubating the enzyme at the pH range of 1.0–11.0 at room temperature for 1 h and assayed at 37 ℃. **B** The effect of temperature on prolyl endopeptidase activity and stability. The activity for optimum temperature was determined between 20–80 ℃ at pH 4.0. The temperature stability was analyzed by incubating the enzyme for 1 h at the temperature range of 4–80 ℃. **C** The effect of various metals concentration on recombinant prolyl endopeptidase activity. The recombinant protein activity was analyzed after incubating with various metals at room temperature for 1 h. (*p < 0.05, **p < 0.01, ****p < 0.0001, ns: not significant). **D** The effect of alcohol on the activity of recombinant prolyl endopeptidase. Following the treatment with different concentrations of alcohol at 37 ℃ for 8 h, the residue activity of prolyl endopeptidase was determined at 37 ℃ and pH 4.0. All experiments were conducted in triplicate

The effects of pH and temperature on the activity and stability of rAN-PEP using Z-Gly-Pro-pNA as substrate are shown in Fig. 3. According to the pH profile, the enzyme showed the highest activity at pH 4.0, which is similar to the prolyl endopeptidases reported from *A. niger* [1, 43]. As for the pH stability of recombinant prolyl endopeptidase, over 90% of its maximal activity was retained within a pH range of 1.0–8.0 (Fig. 3A). rAN-PEP retained high activity at a pH of 5.3–5.6, which is the typical beer brewing pH, suggesting the potential application of recombinant prolyl endopeptidase in beer production. This is different from other prolyl endopeptidases from fungi and bacteria [7, 32–34], which work well at the pH range of 7.0–8.0. The AN-PEP expressed in *T. reesei* presented its maximum activity at 60 °C (Fig. 3B), which approaches the highest optimum temperature (63 °C) for the prolyl endopeptidase from *Sphaerobacter thermophile* [44]. The stability analysis revealed that rAN-PEP showed almost no loss of activity after incubation at 20–40 °C for 60 min, and the activity decreased rapidly when the incubation temperature was above 50 °C (Fig. 3B). Importantly, the rAN-PEP also retained full activity at temperatures below 20 °C, which is the common temperature during beer brewing. As shown in Additional file 1: Fig. S2A, 50% of the rAN-PEP enzyme activity was retained after 4 h of incubation at 50 °C, whereas the enzyme activity was completely abolished at a higher temperature (55 °C). These results indicated that the temperature stability of prolyl endopeptidase from *A. niger* in this study resembles that from *A. oryzae* [35].

The sensitivity of recombinant prolyl endopeptidase to various metal ions, SDS, PMSF, and EDTA is shown in Additional file 1: Fig. S2B. At the concentration of 5 mM, the metal ions Zn^2+^, Mn^2+^, Cu^2+^, Co^2+^, Mg^2+^, and K^+^ had no significant effect (less than 30% change in relative enzyme activity) on the enzyme activity. In contrast, 5 mM Na^+^, Ni^2+^, Li^+^, and Fe^2+^ showed an activating effect on enzyme activity, while Fe^3+^ showed negative effects. Consistent with the previous research [28], the rAN-PEP enzyme activity was inhibited by PMSF and EDTA. rAN-PEP showed the sensitivities to the concentration of Fe^2+^ but seemed unaffected by the concentrations of Mg^2+^ and Fe^3+^(Fig. 3C). Considering the application of prolyl endopeptidase in beer, the effect of alcohol on rAN-PEP activity was also investigated. Surprisingly, the recombinant rAN-PEP retained 97% activity under the condition of 10% alcohol (Fig. 3D), which provides the possibility for the application of AN-PEP in beer brewing.

### The production of gluten-free beer

**Fig. 4:**
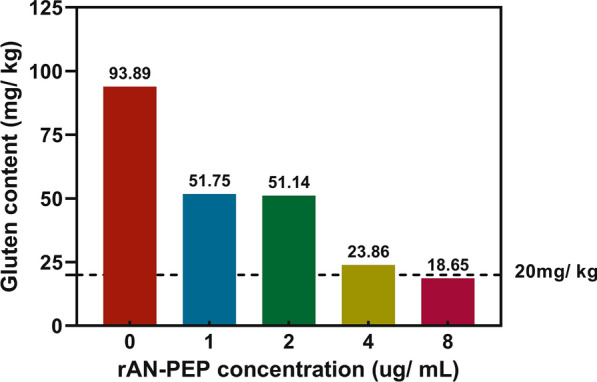
Analysis of gluten content in maturated beer treated with different concentrations of recombinant prolyl endopeptidase (rAN-PEP). The dotted line indicates the Codex threshold for gluten-free food

Gluten refers to a complex class of alcohol-soluble fractions present in the endosperm of wheat, barley, rye, and their crossbred varieties, which causes the haze in beer. Haze can occur at different stages during beer brewing, which is the result of interaction between polyphenolic procyanidins and beer proteins like proline-rich gluten. Gluten also triggers an autoimmune response in susceptible individuals with celiac disease and other gluten-related disorders [45, 46]. A lifelong gluten-free diet, including gluten-free beer, is currently the only effective treatment for susceptible individuals [47]. To produce gluten-free beer, we used different concentrations of rAN-PEP suspension to treat malt wort supplemented with hop and yeast and measured the gluten content using RIDASCREEN^®^ Gliadin Competitive when beer was maturated. As shown in Table 1, only a small rAN-PEP suspension (approximately 2.5 mg/L) can efficiently degrade all gluten during beer fermentation, making the gluten level in beer below the detection limit (10 mg gluten/kg beer), which is lower than the threshold (20 mg of gluten/kg foods) for gluten-free products [29]. In addition, adding the rAN-PEP to the maturated beer without rAN-PEP treatment can exhaustively hydrolyze all gluten (Fig. 4) at a colder temperature (9 ℃), which is a common temperature for beer storage. Total polyphenol content in the rAN-PEP-treated beer (Beer-9 in Table 1) was lower than that in the untreated one (reference beer, Beer-0-1 in Table 1), and the latter looks cloudy in appearance (Additional file 1: Fig. S3). In this study, we demonstrated that rAN-PEP exhibited excellent performance toward hydrolyzing the gluten in beer.

**Table 1:** Preparation of gluten-free beer with the recombinant prolyl endopeptidase

Number	Treatment	The concentration of rAN-PEP (ug/ mL)	Mature beer quality parameters		
			pH	Total protein (ug/ mL)	Gluten level (mg/ kg)
Beer-0–1	Blank control	–	4.59	309.79	82.07
Beer-0–2		–	4.73	328.04	84.32
Beer-0–3		–	4.69	325.93	83.67
Beer-1	Crude rAN-PEP supernatant	2.50	4.56	229.79	< 10.00
Beer-2		5.00	4.54	215.40	< 10.00
Beer-3		10.00	4.57	194.35	< 10.00
Beer-4		20.00	4.52	171.19	< 10.00
Beer-5		40.00	4.50	136.46	< 10.00
Beer-6		80.00	4.62	138.91	< 10.00
Beer-7		120.00	4.73	173.30	< 10.00
Beer-8	Negative control	–	4.46	197.86	82.22
Beer-9	Purified rAN-PEP	2.50	4.36	168.74	< 10.00

## Conclusions

Our research demonstrated that strong functional expression of AN-PEP could be achieved through recombinant expression in *T. reesei*, and corn cob as an alternative cheaper and renewable carbon source showed great potential toward boosting rAN-PEP production. Meanwhile, the obtained rAN-PEP exhibited excellent performance toward hydrolyzing the gluten in beer and gluten-free beer brewing. In summary, this research offers a more promising approach for the industrial production of AN-PEP and lays the foundation for the availability of cost-effective and sustainable AN-PEP and thus the widespread application in the food and pharmaceutical fields.

## Supplementary Information

**Additional file 1:** **Table. S1.** Primers used in this study.^a^: The underlined sequences indicate the overlapping bases between the target gene and vector for constructing the recombinant expression plasmid. **Table. S2** Purification of recombinantly expressed *A. niger* prolyl endopeptidase in *T. reesei*. **Fig.** **S1.** SDS-PAGE analysis of curde rAN-PEP and purified rAN-PEP from *T. reesei*. M, protein marker; Negative control, the supernatant from *T. reesei* TU6. **Fig. S2.** Effects of temperature and metal ions on recombinant prolyl endopeptidase. (A) The residue prolyl endopeptidase activity was determined 1 h following incubation at 40 ℃, 50 ℃, or 55 ℃. (B) The recombinant prolyl endopeptidase was analyzed by incubating with 5 mM various metal ions for 1 h. The metal ions marked with a red arrow were further treated with recombinant protein at various concentration as shown in Figure 3C. All the experiments were conducted in triplicate (****p<0.0001). **Fig. S3.** Total polyphenols testing as well as the appearance of gluten-free beer. (A) Total polyphenols of reference beer (-rAN-PEP) and rAN-PEP-treated beer (+rAN-PEP) were measured using a spectrophotometer. All the experiments were conducted in triplicate (****p<0.0001). (B) The difference in appearance of the reference beer and rAN-PEP-treated beer is shown in the picture

## Data Availability

All data generated or analyzed during this study are included in this published article and its additional files.

## Abbreviations

AN-PEP: Prolyl endopeptidase from *Aspergillus niger*
rAN-PEP: Recombinant AN-PEP in this study
CD: Celiac disease
CBHI: Cellobiohydrolase I
GRAS: Generally regarded as safe
FDA: The United States Food and Drug Administration
MM: Minimal medium
PDA: Potato dextrose agar
Z-Gly-Pro-*p*NA: Benzyloxycarbonyl-glycine-proline-*p*-nitroanilide
PMSF: Phenylmethylsulfonyl fluoride
SDS: Sodium dodecyl sulfate
EDTA: Ethylene diamine tetraacetic acid
AOAC: Association of Official Analytical Chemists
OMA: Official method of analysis
LOD: The limit of detection
LOQ: The limit of quantification
