# Supplementary material for: High-level production of Aspergillus niger prolyl endopeptidase from agricultural residue and its application in beer brewing
Source: Microb Cell Fact. 2023 May 4;22:93. doi: 10.1186/s12934-023-02087-1 (PMC10161650; doi:10.1186/s12934-023-02087-1)

**Additional file 1**

**High-level production of Aspergillus niger prolyl endopeptidase from agricultural residue and its application in beer brewing**

Minglu Liu^1,2^, Meng Hu^1^, Hui Zhou^1,2^, Zhiyang Dong^1,*^, Xiuzhen Chen^1,*^

^1^. *State Key Laboratory of Microbial Resources, Institute of Microbiology, Chinese Academy of Sciences, Beijing, 100101, China*

^2^. *University of Chinese Academy of Sciences, Beijing, 100049, China*

^*^ Corresponding author

Xiuzhen Chen: [chenxiuzhen@im.ac.cn](mailto:chenxiuzhen@im.ac.cn), +86-10-64807331

Zhiyang Dong: [dongzy@im.ac.cn](mailto:dongzy@im.ac.cn), +86-10-64807337

Supplementary Table. S1. Primers used in this study.

| **Primers** | **Sequences (5’-3’) ^a^** | **Target** |
| --- | --- | --- |
| FAN-PEP | TTCTTGGCCACAGCTCGTGCTGCTCGCCCCCGTCTTGTG | Construction expression plasmid pCBH12-AnPEP and pCBH12-AnPEP* |
| RAN-PEP | AGCCCGGTCACGAAAGCCTCAAGCATAATACTCCTCCACCCACTCC |  |
| Fcbh12 | GGCTTTCGTGACCGGGCTTC |  |
| Rcbh12 | AGCACGAGCTGTGGCCAAGAA |  |
| Fvector | CAGCAGATGATAATGATTCCGCTCTAGAAAGGGCAGCTTCAATTCGC | Construction expression vector pCBH12 |
| Rvector | TGATACACACAAGTCTGCCAGTCTAGAAAGGGCAGCTTGGCGTAATC |  |
| Fcbh1 | GGTTTGGATGCAGTTGTCGACCGTGGCTCACCGAAAAGCAAG |  |
| Rcbh1 | TGAAGCCCGGTCACGAAAGCCAGCACGAGCTGTGGCCAAGAA |  |
| Fcbh2 | GGCTTTCGTGACCGGGCTTCA |  |
| Rcbh2 | GCGGAATCATTATCATCTGCTGCCG |  |
| Fpyr4 | CTGGCAGACTTGTGTGTATCATTCAC |  |
| Rpyr4 | GTCGACAACTGCATCCAAACCATC |  |
| F-identify | AGATAGCCTCATTAAACGGAATGAGCT | Verify the chromosomal integration of PEP expression cassette |
| R-identify | CTCACTAGCTACTGATCGACAAAGACC |  |

^a^: The underlined sequences indicate the overlapping bases between the target gene and vector for constructing the recombinant expression plasmid.

Supplementary Table. S2 Purification of recombinantly expressed *A. niger* prolyl endopeptidase in *T. reesei*.

| **Purification of steps** | **Volume (mL)** | **Volumetric activity (U/ mL)** | **Protein concentration (mg/ mL)** | **Total activity (U)** | | **Total protein (mg)** | | **Specific activity (U/ mg)** | | **Purification factor (fold)** | **Yield (%)** |
| --- | --- | --- | --- | --- | --- | --- | --- | --- | --- | --- | --- |
| Crude culture supernatant | 48 | 26.833 | 0.804 | 1287.984 | | 38.592 | | 33.374 | 1.00 | | 100.00 |
| Protein precipitated with ammonium sulfate after dialysis | 15 | 59.670 | 0.618 | 895.056 | 9.266 | | 96.601 | | 2.89 | | 69.49 |
| Amicon Ultra 30,000 MWCO Membrane | 10.5 | 63.299 | 0.339 | 664.640 | 3.560 | | 186.697 | | 5.59 | | 51.60 |
| HiTrap DEAE FF column | 2.5 | 231.742 | 1.076 | 579.355 | 2.690 | | 215.374 | | 6.45 | | 44.98 |

Supplementary Fig. S1. SDS-PAGE analysis of curde rAN-PEP and purified rAN-PEP from *T. reesei*. M, protein marker; Negative control, the supernatant from *T. reesei* TU6.


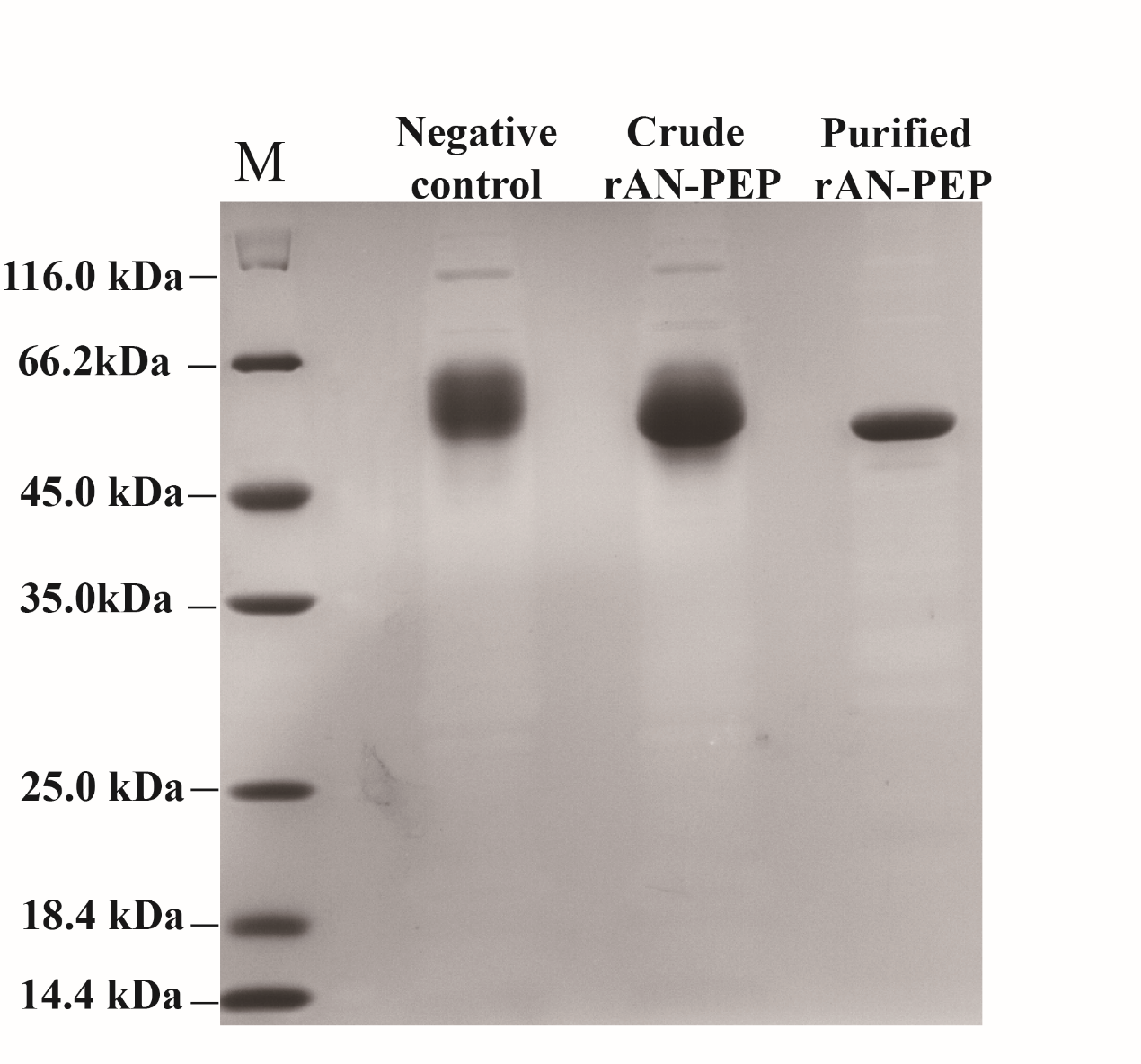


Supplementary Fig. S2. Effects of temperature and metal ions on recombinant prolyl endopeptidase. (A) The residue prolyl endopeptidase activity was determined 1 h following incubation at 40 ℃, 50 ℃, or 55 ℃. (B) The recombinant prolyl endopeptidase was analyzed by incubating with 5 mM various metal ions for 1 h. The metal ions marked with a red arrow were further treated with recombinant protein at various concentration as shown in Figure 3C. All the experiments were conducted in triplicate (*****p*<0.0001).


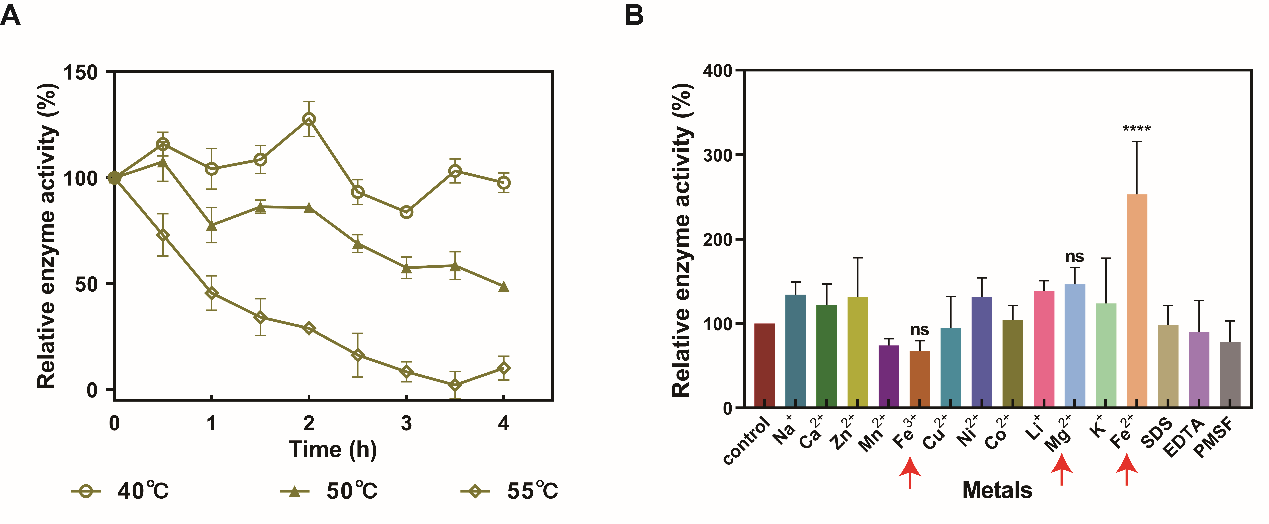


Supplementary Fig. S3. Total polyphenols testing as well as the appearance of gluten-free beer. (A) Total polyphenols of reference beer (-rAN-PEP) and rAN-PEP-treated beer (+rAN-PEP) were measured using a spectrophotometer. All the experiments were conducted in triplicate (*****p*<0.0001). (B) The difference in appearance of the reference beer and rAN-PEP-treated beer is shown in the picture.


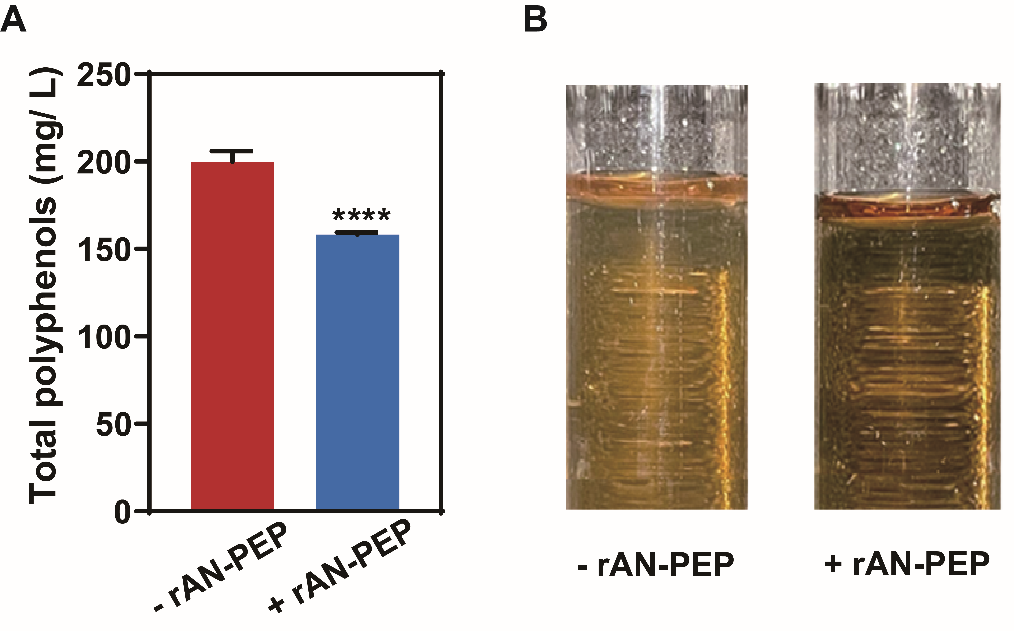

Supplement: Supplementary file 1 — Additional file 1: Table. S1. Primers used in this study.a: The underlined sequences indicate the overlapping bases between the target gene and vector for constructing the recombinant expression plasmid. Table. S2 Purification of recombinantly expressed A. niger prolyl endopeptidase in T. reesei. Fig. S1. SDS-PAGE analysis of curde rAN-PEP and purified rAN-PEP from T. reesei. M, protein marker; Negative control, the supernatant from T. reesei TU6. Fig. S2. Effects of temperature and metal ions on recombinant prolyl endopeptidase. (A) The residue prolyl endopeptidase activity was determined 1 h following incubation at 40 ℃, 50 ℃, or 55 ℃. (B) The recombinant prolyl endopeptidase was analyzed by incubating with 5 mM various metal ions for 1 h. The metal ions marked with a red arrow were further treated with recombinant protein at various concentration as shown in Figure 3C. All the experiments were conducted in triplicate (****p<0.0001). Fig. S3. Total polyphenols testing as well as the appearance of gluten-free beer. (A) Total polyphenols of reference beer (-rAN-PEP) and rAN-PEP-treated beer (+rAN-PEP) were measured using a spectrophotometer. All the experiments were conducted in triplicate (****p<0.0001). (B) The difference in appearance of the reference beer and rAN-PEP-treated beer is shown in the picture [file 12934_2023_2087_MOESM1_ESM.docx]
